# Editorial: Accessory Cells of Sensory Systems and Their Functional Roles

**DOI:** 10.3389/fnins.2022.965580

**Published:** 2022-06-30

**Authors:** Sarah D. Ackerman, Aakanksha Singhvi, Laura Bianchi

**Affiliations:** ^1^Department of Pathology and Immunology, Washington University School of Medicine, Saint Louis, MO, United States; ^2^Division of Basic Sciences, Fred Hutchinson Cancer Center, Seattle, WA, United States; ^3^Department of Physiology and Biophysics, University of Miami Miller School of Medicine, Miami, FL, United States

**Keywords:** glia, accessory cells, sensory, touch, hearing, smell, sight

## Introduction

Humans experience the world through the five primary senses: sight, touch, hearing, smell, and taste, most of which are conserved across the animal kingdom. Acute loss of one of these senses can be disorienting and prolonged sensory impairments can greatly impact one's quality of life. Environmental stimuli are received by specialized sensory structures innervated by neurons to elicit an appropriate behavioral response (Roper and Chaudhari, 2017; Seabrook et al., 2017; Driver and Kelley, 2020; Ray and Singhvi, 2021), for example, the retraction of a hand from a hot or freezing surface. Sensory neurons are accompanied by a diverse group of non-neuronal cells including glia. Accessory cells/glia are essential regulators of neuronal health through direct metabolic coupling and through careful maintenance of ionic balance at peripheral and central synapses (Ray and Singhvi, 2021). An increasing body of evidence has revealed that glia are not only “support” cells, but directly influence circuit architecture and signaling (Lago-Baldaia et al., 2020; Perez-Catalan et al., 2021; Ray and Singhvi, 2021). In this issue, “*Accessory Cells of Sensory Systems*,” new studies highlight the importance of non-neuronal cells to *Sight, Touch, Hearing*, and *Smell*, from phagocytic function following injury, to tuning of neuronal activity.

## Sight

Müller cells are a major type of glial cell in the retina, and they are responsible for homeostatic and metabolic support of retinal cells (Reichenbach and Bringmann, 2013). In particular, Müller cells mediate transcellular ion, water and bicarbonate transport, glutamate uptake, and they provide trophic and anti-oxidative support to the cells of the retina. In addition, Müller cells guide light through the inner retinal tissue, thus enhancing the signal/noise ratio (Franze et al., 2007). In disease conditions, a subset of Müller cells may differentiate into neuronal progenitor cells, thereby giving rise to new photoreceptors and neurons (Das et al., 2006; Bernardos et al., 2007; Roesch et al., 2008). Müller cells also have phagocytotic functions (Long et al., 1986).

In this issue, Lew et al. investigated the molecular underpinnings of Müller cell-mediated phagocytosis, a process still largely uncharacterized, and its consequences on photoreceptor degeneration. Using in culture phagocytosis assays, two distinct *in vivo* models, western blots, and immunocytochemistry, the authors demonstrate that phagocytosis of photoreceptor outer segment fragments (POS) by human and murine Müller cells is mediated by the TAM engulfment receptor tyrosine kinase MERTK, which has been implicated also in Retinal Pigmented Epithelium (RPE)-mediated phagocytosis (Gal et al., 2000; Parinot and Nandrot, 2016). The authors also show that MERTK utilizes both galactin-3 and protein S as its ligands. These two ligands most likely share the same binding site because their effect on phagocytosis is not additive. Knockout of *mertk* or of the gene encoding galactin-3, *lgals3*, lead to degeneration of photoreceptors by postnatal day 35 and activation of the Müller cells as measured by Glial fibrillary acidic protein (GFAP) expression. Interestingly, *mertk*^−/−^ and *lgals3*^−/−^ double knockout mice show significantly enhanced Müller cell activation and photoreceptor degeneration as compared to the single knockouts. The authors exclude the involvement of microglia in this effect and thus conclude that it must be mediated by the Müller cells themselves. To conclude, this study identifies galactin-3 acting on Müller glial cells, as a protective factor in photoreceptor degeneration. Notably, previous studies showed that microglia activation is reduced in galactin-3-defective models, highlighting that galactin-3 may have opposite effects on microglia vs. Müller cells. This urges the consideration of cell-specific approaches for the studies of these mechanisms and for the identification of future therapeutical approaches.

## Touch

Four of the five types of touch receptors embedded in the human skin are composed of nerve endings and accessory cells. These include the Pacinian, Meissner, and Ruffini corpuscles, and the Merkel disks. In addition, the Krause bulbs, which detect vibrations in addition to cold temperature, are also composed of nerve endings and accessory cells. On the other hand, the free nerve endings that detect temperature, painful stimuli, and light touch, are unencapsulated dendrites of sensory neurons voided of any type of accessory cells. In a review published in this issue, Suazo et al. update us on what is known about the anatomy, development, as well as function of Meissner and Pacinian corpuscles that detect light touch and vibration, respectively, primarily focusing on their accessory cells. Indeed, in both the Meissner and Pacinian corpuscles, flattened Schwann cells form lamellae organized in parallel (Meissner) and concentric (Pacinian) structures around the nerve terminal. Both corpuscles also have outer capsules composed of perineurial fibroblast-like cells and contain a complex extracellular matrix. While initially the lamellae of the Meissner and Pacinian corpuscles were thought to primarily provide structural integrity to the corpuscles, their cellular and functional features suggest that they have major roles in the transduction of touch. Indeed, recent work on the Meissner and Pacinian corpuscles from the duck bill (called Grandry's and Herbtst corpuscles, respectively, in the duck) by the lab of Bagriantsev has shown that these cells are endowed with mechanically gated ion channels. The lamellae of the Meissner corpuscles also express R-type voltage gated calcium channels and can fire action potentials when stimulated by current injection or by mechanical forces (Nikolaev et al., 2020; Ziolkowski et al., 2022). These exciting new data not only support that these cells are intrinsically mechanosensitive, but also that they can propagate this information potentially to other cells. Intriguingly, electron micrographs of the lamellar cells from the cat show that they have clear core vesicles and electron dense regions near the plasma membrane reminiscent of synapses (Pawson et al., 2009). Furthermore, lamellar cells from the Pacinian corpuscles of the cat express synaptic proteins synaptobrevin VAMP2 and SNAP-23 (Pawson et al., 2009). Pawson and colleagues using electrophysiological techniques on the Pacinian corpuscles of the cat, demonstrated that GABA released by the lamellae inhibits action potentials in the nerve ending during the static portion of the indentation (Pawson et al., 2009). They also showed that glutamate released by the nerve ending is responsible for action potentials during the static portion of the indentation and that most likely induces GABA release from the lamellar cells (Pawson et al., 2007, 2009). These older data combined with new results obtained from Meissner and Pacinian corpuscles of the duck paint a picture of a complex cross talk between accessory cells and nerve endings in these corpuscles that might be crucial for touch sensation. Intriguingly, a similar mechanism of cross talk and analogous mechanosensitivity of accessory cells have been described for the nose touch receptors of the nematode *C. elegans*, suggesting conservation of function across species (Fernandez-Abascal et al., 2022). Future studies leveraging the power of genetics in the duck and in invertebrate models should help dissect this mechanism of cross talk even further and should identify the mechanosensitive channels expressed in the lamellar cells.

## Hearing

Hearing is an essential sense that not only allows us to enjoy the world around us, but also to navigate our environment with high precision. The sensory organ required for proper hearing is called the organ of Corti, housed in the cochlea (inner ear). In the cochlea, sound vibrations activate mechanosensory cells called hair cells, which decode vibrational information (sound) to precisely stimulate the auditory nerve and in turn, auditory cortex. Proper signaling within auditory circuits requires a variety of both peripheral and central glia, including Support Cells within the organ of Corti, Schwann cells, Satellite Glia, Oligodendrocytes, and Astrocytes (Kohrman et al., 2021). Another prominent, non-neuronal cell population within the cochlea are tissue-resident macrophages, which are thought to carry out homeostatic roles in healthy tissues (Hough et al., 2022).

Chronic exposure to loud noises can lead to long-term hearing loss due to loss or dysfunction of hair cells and associated glia (astrocytes and microglia). In addition, deleterious sound is followed by a robust increase in macrophage populations within the cochlea (Hirose et al., 2005). Shortly following noise exposure, immune cell engulfment of damaged hair cells is important for cochlear health; however, long-term inflammation can ultimately lead to hearing loss (He et al., 2020). In this issue, Shin et al. characterize the source and trajectory of cochlear immune cells in a mouse model of acoustic overstimulation. Using RNAseq, they find that the pro-inflammatory cytokine *Ccl2* is upregulated in the cochlea a mere 3 h following noise exposure. Within 1–2 days post noise, peripherally-derived monocytes infiltrate the cochlea, where they subsequently transform into macrophages. Importantly, depletion of monocytes (and thus macrophages) following overstimulation does not prevent hearing loss, consistent with the early requirement of macrophages in debris clearance (Bae et al., 2021). Together, these findings suggest that anti-inflammatory therapies to prevent noise induced hearing loss must be temporally restricted.

## Smell

Olfaction, the perception of volatile chemosensory cues, is one of the most ancient sensory modalities. Olfactory sensory neurons sense odor molecules at either the olfactory epithelium in vertebrates, or in specialized sensory units in invertebrates, called sensilla (Vosshall, 2000; Buck, 2004). These anatomical structures house both neurons and glia. The underlying sensory neuron transduction machinery that transmits odor information has been exquisitely dissected (Shirsat and Siddiqi, 1993; Wilson, 2013), and glia have been shown to secrete odor binding proteins and clearance enzymes (Sun et al., 2018). However, how sense-organ glia impact olfactory perception is only recently being explored (Bianchi, 2020; Duan et al., 2020).

*Drosophila* sense odorants at structures on their antenna called sensilla, that house 2–4 sensory neurons and three support cell sub-types called trichogen, thecogen, and tormogen (Sen et al., 2005). Using genetically encoded sensors for K^+^ and Ca^2+^ expressed in different support cells, Prelic et al. uncovered active cellular responses in glia. First, they observed that thecogen and tormogen glia exhibit distinct patterns of ion fluxes upon presentation of the odor proxy VUAA1, concomitant with neuron activation. Next, by genetically ablating thecogen glia in adults, they found that individual sensory units exhibit altered mechanical sensitivity without alteration of resting neuron activity. This suggests the intriguing notion that these glia may contribute to odor processing by aiding detection of spatiotemporal odorant pulses as would be experienced during the air turbulence experienced during the animal flight. These findings parallel recent work in *C. elegans* showing glial responses to odorants (Duan et al., 2020).

## Conclusion

Emerging studies, including those described above, reveal more active participation by glia and other accessory cells in sensory perception than previously appreciated. Mechanistically, these cells do so by phagocytosis of sensory endings and cells (Raiders et al., 2021; Lew et al.), modulation of ionic milieu (Ray and Singhvi, 2021), and release of neuromodulators (Pawson et al., 2007, 2009; Duan et al., 2020; Fernandez-Abascal et al., 2022). This is of note since at least in *C. elegans*, glia can respond to diverse external stimuli independent of neurons (Procko et al., 2011; Duan et al., 2020; Fernandez-Abascal et al., 2022). How different glia across sensory systems regulate these functions in response to sensory cues remains largely a mystery to date, presenting a rich avenue for future inquiry. We propose that an understanding of how sensory percepts are processed in the periphery in health or sensory disorders requires not only a dissection of how a sensory cell or neuron functions, but rather a composite study of how sense-organ glia-neuron units coordinately respond to environmental cues.

## Author Contributions

SDA wrote the Hearing and Introduction sections. AS wrote the Smell and Conclusion sections. LB wrote the Sight and Touch sections, and curated literature citations and final edits. All authors contributed to the article and approved the submitted version.

## Funding

Work in the Bianchi's laboratory is supported by NIH grants (NS106951, NS10561, and NS127146). Work in the Ackerman laboratory is supported by the NIH grant (NS121137). Work in the Singhvi lab is supported by NIH grant (NS 114222).

## Conflict of Interest

The authors declare that the research was conducted in the absence of any commercial or financial relationships that could be construed as a potential conflict of interest.

## Publisher's Note

All claims expressed in this article are solely those of the authors and do not necessarily represent those of their affiliated organizations, or those of the publisher, the editors and the reviewers. Any product that may be evaluated in this article, or claim that may be made by its manufacturer, is not guaranteed or endorsed by the publisher.

## References

[B1] Bae S. H. Yoo J. E. Hong J. W. Park H. R. Noh B. Kim H.. (2021). LCCL peptide cleavage after noise exposure exacerbates hearing loss and is associated with the monocyte infiltration in the cochlea. Hear. Res. 412, 108378. 10.1016/j.heares.2021.108378 34735822

[B2] Bernardos R. L. Barthel L. K. Meyers J. R. Raymond P. A. (2007). Late-stage neuronal progenitors in the retina are radial Muller glia that function as retinal stem cells. J. Neurosci. 27, 7028–7040. 10.1523/JNEUROSCI.1624-07.2007 17596452 PMC6672216

[B3] Bianchi L. (2020). *C. elegans* glia are bona fide odorant receptor cells. Neuron 108, 588–589. 10.1016/j.neuron.2020.10.026 33242427

[B4] Buck L. B. (2004). Olfactory receptors and odor coding in mammals. Nutr. Rev. 62, S184–188; discussion S224-141. 10.1111/j.1753-4887.2004.tb00097.x 15630933

[B5] Das A. V. Mallya K. B. Zhao X. Ahmad F. Bhattacharya S. Thoreson W. B.. (2006). Neural stem cell properties of Muller glia in the mammalian retina: regulation by Notch and Wnt signaling. Dev. Biol. 299, 283–302. 10.1016/j.ydbio.2006.07.029 16949068

[B6] Driver E. C. Kelley M. W. (2020). Development of the cochlea. Development 147, dev162263. 10.1242/dev.162263 32571852 PMC7327997

[B7] Duan D. Zhang H. Yue X. Fan Y. Xue Y. Shao J.. (2020). Sensory glia detect repulsive odorants and drive olfactory adaptation. Neuron 108, 707 e708–721 e708. 10.1016/j.neuron.2020.08.026 32970991

[B8] Fernandez-Abascal J. Johnson C. K. Graziano B. Wang L. Encalada N. Bianchi L. (2022). A glial ClC Cl(-) channel mediates nose touch responses in *C. elegans*. Neuron 110, 470 e477–485 e477. 10.1016/j.neuron.2021.11.010 34861150 PMC8813913

[B9] Franze K. Grosche J. Skatchkov S. N. Schinkinger S. Foja C. Schild D.. (2007). Mand#xfc;ller cells are living optical fibers in the vertebrate retina. Proc. Natl. Acad. Sci. U.S.A. 104, 8287–8292. 10.1073/pnas.0611180104 17485670 PMC1895942

[B10] Gal A. Li Y. Thompson D. A. Weir J. Orth U. Jacobson S. G.. (2000). Mutations in MERTK, the human orthologue of the RCS rat retinal dystrophy gene, cause retinitis pigmentosa. Nat. Genet. 26, 270–271. 10.1038/81555 11062461

[B11] He W. Yu J. Sun Y. Kong W. (2020). Macrophages in noise-exposed cochlea: changes, regulation and the potential role. Aging Dis. 11, 191–199. 10.14336/AD.2019.0723 32010492 PMC6961779

[B12] Hirose K. Discolo C. M. Keasler J. R. Ransohoff R. (2005). Mononuclear phagocytes migrate into the murine cochlea after acoustic trauma. J. Comp. Neurol. 489, 180–194. 10.1002/cne.20619 15983998

[B13] Hough K. Verschuur C. A. Cunningham C. Newman T. A. (2022). Macrophages in the cochlea; an immunological link between risk factors and progressive hearing loss. Glia 70, 219–238. 10.1002/glia.24095 34536249

[B14] Kohrman D. C. Borges B. C. Cassinotti L. R. Ji L. Corfas G. (2021). Axon-glia interactions in the ascending auditory system. Dev. Neurobiol. 81, 546–567. 10.1002/dneu.22813 33561889 PMC9004231

[B15] Lago-Baldaia I. Fernandes V. M. Ackerman S. D. (2020). More than mortar: glia as architects of nervous system development and disease. Front. Cell Dev. Biol. 8, 611269. 10.3389/fcell.2020.611269 33381506 PMC7767919

[B16] Long K. O. Fisher S. K. Fariss R. N. Anderson D. H. (1986). Disc shedding and autophagy in the cone-dominant ground squirrel retina. Exp. Eye Res. 43, 193–205. 10.1016/S0014-4835(86)80087-2 3758219

[B17] Nikolaev Y. A. Feketa V. V. Anderson E. O. Schneider E. R. Gracheva E. O. Bagriantsev S. N. (2020). Lamellar cells in Pacinian and Meissner corpuscles are touch sensors. Sci. Adv. 6, abe6393. 10.1126/sciadv.abe6393 33328243 PMC7744075

[B18] Parinot C. Nandrot E. F. (2016). A comprehensive review of mutations in the MERTK proto-oncogene. Adv. Exp. Med. Biol. 854, 259–265. 10.1007/978-3-319-17121-0_35 26427420

[B19] Pawson L. Pack A. K. Bolanowski S. J. (2007). Possible glutaminergic interaction between the capsule and neurite of Pacinian corpuscles. Somatosens. Mot. Res. 24, 85–95. 10.1080/08990220701388364 17558925

[B20] Pawson L. Prestia L. T. Mahoney G. K. Guclu B. Cox P. J. Pack A. K. (2009). GABAergic/glutamatergic-glial/neuronal interaction contributes to rapid adaptation in pacinian corpuscles. J. Neurosci. 29, 2695–2705. 10.1523/JNEUROSCI.5974-08.2009 19261864 PMC2727419

[B21] Perez-Catalan N. A. Doe C. Q. Ackerman S. D. (2021). The role of astrocyte-mediated plasticity in neural circuit development and function. Neural Dev. 16, 1. 10.1186/s13064-020-00151-9 33413602 PMC7789420

[B22] Procko C. Lu Y. Shaham S. (2011). Glia delimit shape changes of sensory neuron receptive endings in *C. elegans*. Development 138, 1371–1381. 10.1242/dev.058305 21350017 PMC3050665

[B23] Raiders S. Han T. Scott-Hewitt N. Kucenas S. Lew D. Logan M. A.. (2021). Engulfed by glia: glial pruning in development, function, and injury across species. J. Neurosci. 41, 823–833. 10.1523/JNEUROSCI.1660-20.2020 33468571 PMC7880271

[B24] Ray S. Singhvi A. (2021). Charging up the periphery: glial ionic regulation in sensory perception. Front. Cell Dev. Biol. 9, 687732. 10.3389/fcell.2021.687732 34458255 PMC8385785

[B25] Reichenbach A. Bringmann A. (2013). New functions of Muller cells. Glia 61, 651–678. 10.1002/glia.22477 23440929

[B26] Roesch K. Jadhav A. P. Trimarchi J. M. Stadler M. B. Roska B. Sun B. B.. (2008). The transcriptome of retinal Muller glial cells. J. Comp. Neurol. 509, 225–238. 10.1002/cne.21730 18465787 PMC2665263

[B27] Roper S. D. Chaudhari N. (2017). Taste buds: cells, signals and synapses. Nat. Rev. Neurosci. 18, 485–497. 10.1038/nrn.2017.68 28655883 PMC5958546

[B28] Seabrook T. A. Burbridge T. J. Crair M. C. Huberman A. D. (2017). Architecture, function, and assembly of the mouse visual system. Annu. Rev. Neurosci. 40, 499–538. 10.1146/annurev-neuro-071714-033842 28772103

[B29] Sen A. Shetty C. Jhaveri D. Rodrigues V. (2005). Distinct types of glial cells populate the *Drosophila antenna*. BMC Dev. Biol. 5, 25. 10.1186/1471-213X-5-25 16281986 PMC1310525

[B30] Shirsat N. Siddiqi O. (1993). Olfaction in invertebrates. Curr. Opin. Neurobiol. 3, 553–557. 10.1016/0959-4388(93)90055-4 8219721

[B31] Sun J. S. Xiao S. Carlson J. R. (2018). The diverse small proteins called odorant-binding proteins. Open Biol. 8, 180208. 10.1098/rsob.180208 30977439 PMC6303780

[B32] Vosshall L. B. (2000). Olfaction in *Drosophila*. Curr. Opin. Neurobiol. 10, 498–503. 10.1016/S0959-4388(00)00111-2 10981620

[B33] Wilson R. I. (2013). Early olfactory processing in *Drosophila*: mechanisms and principles. Annu. Rev. Neurosci. 36, 217–241. 10.1146/annurev-neuro-062111-150533 23841839 PMC3933953

[B34] Ziolkowski L. H. Gracheva E. O. Bagriantsev S. N. (2022). Tactile sensation in birds: physiological insights from avian mechanoreceptors. Curr. Opin. Neurobiol. 74, 102548. 10.1016/j.conb.2022.102548 35489134 PMC9167745

